# The Relationship Between Psychological Detachment and Employee Well-Being: The Mediating Effect of Self-Discrepant Time Allocation at Work

**DOI:** 10.3389/fpsyg.2018.02426

**Published:** 2018-12-11

**Authors:** XiaoTian Wang, Aimei Li, Pei Liu, Ming Rao

**Affiliations:** ^1^School of Business Administration, Guangdong University of Finance and Economics, Guangzhou, China; ^2^School of Management, Jinan University, Guangzhou, China

**Keywords:** psychological detachment, self-regulatory resources, self-discrepant time allocation at work, employee well-being, polynomial regression

## Abstract

Although research has demonstrated the benefit of psychological detachment for employee well-being, the explanatory mechanisms related to work behaviors underlying this effect remain underdeveloped. Addressing this research gap, we consider self-discrepant time allocation (preferred–actual allocation) as a mediating mechanism through which psychological detachment affects employee well-being. We hypothesize that psychological detachment is associated with self-discrepant time allocation at work. Specifically, we suggest that employees with low detachment tend to allocate more time than preferred to work activities that demand fewer self-regulatory resources and allocate less time than preferred to activities demanding greater self-regulatory resources. These self-discrepant time allocations at work are associated with employee well-being. Polynomial regression analysis and response surface methodology were used to test the hypotheses. The results, based on a sample of 390 faculty members from 19 universities, showed that, when psychological detachment during weekends is low and self-regulatory resources are insufficient, employees will allocate less time than preferred to work activities that require more self-regulatory resources (i.e., researching activities) during the subsequent work period. Instead, employees tend to allocate more time to activities that require less resources (i.e., teaching activities). These discrepancies between actual and preferred time allocation for work activities, in turn, negatively affect employee well-being and mediate the relationship between psychological detachment and employee well-being.

## Introduction

To stay efficient as well as healthy, employees need to disengage mentally from their jobs and to have some rest after work (Sonnentag and Fritz, 2015; Ragsdale and Beehr, 2016). However, many employees regularly deal with work-related issues during off-work time (e.g., evening hours, weekends) on account of increasing workloads and the prevalence of modern communication technologies (e.g., smartphone, laptop). Should employees cope with work-related matters after leaving the workplace, though? How might this lack of detachment affect employee work behavior in subsequent work periods? Does this issue influence employee well-being within the workplace?

Previous research has highlighted the importance of psychological detachment for well-being, suggesting that it allows employees to replenish psychological resources that have been depleted through dealing with job stressors. Researchers have tested the effect of detachment on various indicators of general well-being (e.g., life satisfaction, vigor, positive and negative affect, somatic symptoms, depression) in both between-person and within-person studies (Sonnentag and Fritz, 2015; Sonnentag et al., 2017). From a between-person perspective, research has shown that employees' detachment during non-work time is positively associated with their life satisfaction (Hahn and Dormann, 2013) and positive affect (Davidson et al., 2010) and negatively associated with somatic symptoms (Moreno-Jiménez et al., 2012), and depression (Hahn et al., 2012). Furthermore, research adopting a within-person perspective reveals that detachment during the evening is positively related to positive affect and vigor at the end of that day (Demerouti et al., 2012; Feuerhahn et al., 2014) and negatively related to fatigue the following morning (Sonnentag et al., 2008).

Despite the mounting empirical evidence showing the direct effect of psychological detachment on well-being, there are still some gaps in our knowledge regarding the relationship between psychological detachment and well-being. First, although employee well-being, which reflects an employee's perceptions and feelings within the workplace, is critical to the maintenance and development of employees and organizations (Zheng et al., 2015), prior studies mainly concentrate on general well-being indicators (e.g., life satisfaction, positive and negative affect, psychological strain) rather than employee well-being in particular (Moreno-Jiménez et al., 2009; Fritz et al., 2010a; Sonnentag and Fritz, 2015). However, general well-being cannot adequately represent well-being at work since the workplace context is far different from general life situations (Zheng et al., 2015). Therefore, to shed more light on the influence of psychological detachment on employees' psychological status at work, research utilizing context-specific concepts and measures of employee well-being is needed. Second, the mechanism underlying the effects of detachment on employee well-being is also far from fully understood. Previous studies have focused on the direct effect of detachment on well-being but have not explicitly addressed the mechanism underlying the relationship between detachment and employee well-being. Thus, opening the “black box” of the detachment process could help us to better understand the effect of detachment on employee well-being (Sonnentag et al., 2017). Finally, the impacts of detachment on work behavior are still unclear. Although analysis of psychological detachment has been proposed in order to obtain a better understanding of the influence of non-work factors on work (Sonnentag and Fritz, 2007), until now, the majority of empirical studies on detachment have focused on well-being and health, while the relationship between detachment and work behavior has rarely been examined (Sonnentag, 2018). Given that psychological reactions have been found to directly bring about behavioral responses in the ensuing time period, psychological detachment during off-job time may directly affect work behavior in the next work period, and work behavior is associated with well-being at work. Thus, work behavior may play a critical role in the relationship between detachment and employee well-being. In work situations, time is a finite resource. Therefore, knowing how to allocate time among different work activities is a critical behavior at work, and may be a key mechanism of the relationship between psychological detachment and employee well-being.

To address these research gaps, based on self-regulation theory, we focus on “self-discrepant time allocation at work” (i.e., the difference between preferred and actual time allocations at work), which reflects the time allocation behavior of employees at work. We propose self-discrepant time allocation at work as a key mediator between detachment and employee well-being. Specifically, we expect that employees who experience low detachment during a weekend will have fewer self-regulatory resources during the following week. Thus, they will be inclined to allocate less time than preferred to work activities that require greater self-regulatory resources (e.g., research activities) and allocate more time than preferred to work activities that require less self-regulatory resources or are restoring (e.g., teaching and service activities) in the following work week. The discrepancy between actual and preferred time allocation at work activities is associated with well-being at work, taking the form of an inverted U-shape curve.

## Theoretical Background and Development of Hypotheses

### Psychological Detachment and Self-Discrepant Time Allocation at Work

#### Psychological Detachment and Self-Regulatory Resource Replenishment

The concept of “psychological detachment”—defined in our study's context as mental and physical distance from work during off-hours—can be demonstrated through an employee not only being involved in work activities, such as continually dealing with unfinished work-related tasks, but also not thinking about work-related issues, such as pondering future tasks (Sonnentag and Fritz, 2007). Employees practicing low detachment continually cope with job stressors (e.g., time pressure, work complexity, and role conflict) and finish jobs during non-work periods (e.g., free evenings, weekends, vacations). In doing so, their personal resources, which had been expended during work time, are further depleted, thereby decreasing occupational health and performance. Although personal resource restoration is a core part of the detachment process, it is difficult to identify which personal resources are crucial; that is, which could be replenished via detachment and functioning at work (Ragsdale and Beehr, 2016; Smit, 2016).

Fortunately, self-regulation theory helps us understand that self-regulatory resources are a key personal resource within the detachment process. The theory specifies that “self-regulatory resources” are limited resources that enable the inhibition, modification, and overriding of spontaneous and automatic responses that would otherwise hinder goal-directed behavior and goal achievement (Baumeister et al., 2007). Self-regulatory resources are depleted when individuals conduct self-regulatory or self-controlling behavior such as maintaining attention, counteracting temptations, persevering with difficult tasks, and making decisions in order to achieve goals (Muraven and Baumeister, 2000; Hagger et al., 2010; Hofmann et al., 2012). Consequently, drawing on self-regulatory resources will decrease subsequent self-regulation/self-control ability. An individual's self-regulation ability returns to a normal level only if their self-regulatory resources are replenished, which can be accomplished through distancing from self-regulation/self-control activities such as work or by engaging in leisure activities and taking breaks (Hagger and Chatzisarantis, 2013; Germeys and De Gieter, 2018).

As an important way of restoring a variety of personal resources by refraining from work, psychological detachment can replenish self-regulatory resources via three approaches. First, high levels of psychological detachment mean not deliberating or dealing with work-related issues. By using this approach, employees can avoid further consuming, conserve remaining, and even replenish self-regulatory resources (Ragsdale and Beehr, 2016; Germeys and De Gieter, 2018). Second, employees can experience high psychological detachment through leisure activities (e.g., watching a movie, playing a basketball game) or private social activities (e.g., enjoying a romantic date, going for dinner with a friend) during off-job time. Engaging in these activities implies that the employee is taking a break or rest, which can restore depleted self-regulatory resources (Tyler and Burns, 2008; ten Brummelhuis and Bakker, 2012). Third, psychological detachment is positively related to positive affect (Davidson et al., 2010), and the latter enhances an employee's self-regulation ability (Tice et al., 2007), which implies a replenishment of self-regulatory resources.

#### Psychological Detachment and Time Allocation

According to the self-regulation theory, the self-regulatory resources that an employee has at the beginning of work influence subsequent work behaviors, such as sustaining attention, overriding obstructions, preventing interference, and persisting in complex tasks, and ultimately influences work-related goal attainment (Beal et al., 2005). “Self-discrepancy time allocation at work,” or the differences between actual time allocation and preferred time allocation to categories of work activities, is a typical work behavior, since time holds a vital role in the work process (Dahm et al., 2015). “Preferred time allocation” is the amount of time that an employee intends to devote to categories of work activities. It demonstrates the behavior employees would like to exhibit at work, and the goal of their work. “Actual time allocation” refers to the amount of time that an employee actually expends at work activities. Notably, “time allocation at work” needs to take into account more than one activity since most work involves multiple undertakings. Consequently, within a fixed total time, spending time on one activity impedes time investment on any other activity.

When psychological detachment is low and self-regulatory resources are insufficient, it is more difficult for an employee to allocate time in line with their preferences. Specifically, such an employee will allocate less time than preferred to work activities that require more self-regulatory resources and will instead allocate more time than preferred to activities that require fewer resources, since total work time is fixed. Work activities that demand more self-regulatory resources include activities that are complicated or contain long-term goals (Lilius, 2012; Dahm et al., 2015). Complicated activities are highly demanding for two reasons. First, completing complex activities requires the promotion of “system 2” resources such as analytical reasoning, making rational choices, and meticulous thinking; these behaviors take up a large amount of self-regulatory resources (Kahneman and Frederick, 2002; Baumeister et al., 2008). Second, complex activities incorporate a high level of information load or diversity, which in turn causes high uncertainty about the various paths toward achieving a goal and the best path with which to achieve it. People need to regulate cognitive processes—which expend self-regulatory resources—to successfully deal with a mass of information and find the right way to achieve a goal among multiple possible paths (Campbell, 1988; Hagger et al., 2010). Long-term goals necessarily take a significant amount of time to attain and for the individual to obtain the associated rewards. During these long periods, the as-yet-unfulfilled goals continuously consume self-regulatory resources by occupying attention, memory, and so on (Smit, 2016). Thus, depleted people are more likely to engage in activities that can be done promptly and offer immediate gratification, in order to avoid further depleting their remaining resources (Masicampo and Baumeister, 2011).

Taking these points together, when psychological detachment is low during off-job periods (e.g., weekends), employees will allocate less time than preferred to work activities that require more self-regulatory resources (e.g., complex or long-term goal-directed work activities) and allocate more time than preferred to work activities that require less resources (easy jobs or those that provide immediate rewards) in subsequent work periods (e.g., the next working week).^1^ Accordingly, we posit:

Hypothesis 1a: As psychological detachment during a weekend decreases, actual time allocated to ensuing work activities that require more self-regulatory resources will be less than preferred.
Hypothesis 1b: As psychological detachment during a weekend decreases, actual time allocated to ensuing work activities that require less self-regulatory resources will be greater than preferred.

#### Self-Discrepant Time Allocation at Work and Employee Well-Being

As noted, self-discrepant time allocation refers to the differences between an employee's preferred and actual time allocations among work activities (Dahm et al., 2015). Compared to actual work time, self-discrepant time allocation can more accurately represent the effects of time allocation on well-being. Since each employee has his or her own preferred time allocations among work activities, determined by their particular goals and hopes (Moen et al., 2008; Sturman and Walsh, 2014), the discrepancies between actual and preferred time allocations (the differences between actualities and goals or hopes), rather than the absolute amount of actual allocated time, are more critical to well-being (Wooden et al., 2009). Previous research further indicates that the effect of discrepancies between actual and preferred time allocations on well-being may take the form of an inverted U-shaped curve. For example, studies have found that life satisfaction is lower for employees with misaligned time allocation, regardless of whether the difference is positive or negative (Wunder and Heineck, 2013). Similarly, prior research has indicated that job stress and work–family conflict increase both when actual allocated time exceeds or is less than preferred time allocation (Sturman and Walsh, 2014).

Based on prior research, the present study focuses on “employee well-being,” a context-specific form of well-being that reflects an employee's cognitive and emotional feelings at their workplace (Zheng et al., 2015), and we postulate that relationships between self-discrepant time allocations and employee well-being takes the form of an inverted U-shaped curve. Specifically, employee well-being decreases as the actual time allocated to categories of work activities is less or more than preferred time. First, when actual allocated time is less than preferred, employee well-being will decrease as actual allocated time becomes discrepant from what is preferred. This can be explained by the impairing of goal attainment and identity deficit. Research on self-regulation and goal attainment demonstrates that goal pursuing and goal attainment—especially in respect to a goal in line with one's values and interests—increases well-being (Zimmerman, 2000; Wrosch et al., 2003; Smith et al., 2007). That is, an individual who persists in pursuing and ultimately fulfilling a goal is likely to feel more satisfied, competent, and autonomous, and this also tends to generate more positive affects, which, in turn, increase well-being (Sheldon and Elliot, 1999). Given that time allocation preferences reflect the time an employee plans to deploy in fulfilling a certain work goal, allocating less time than preferred implies a failure of goal attainment, which, in turn, has a passive impact on employee well-being. Consider that preferred allocated time reflects an employee's ideal work identity, which comprises their hopes, wants, and wishes about work, while actual allocated time reflects the employee's actual work identity. An employee may experience an “identity deficit,” or an uncomfortable feeling that their sense of self is challenged, since they have failed to realize an ideal work identity (Pratt and Dutton, 2000). This identity deficit has a passive influence on employee well-being (Pratt, 2000). Second, when actual allocated time exceeds the preferred amount, employee well-being will decrease, following the same pattern as when actual is less than preferred. Most work involves more than one work activity, while work hours are constant in a certain work period (e.g., 8 h per day), and therefore spending time on one activity impedes time available for any other activity (Dahm et al., 2015). Furthermore, although allocating more time than preferred on one work activity can help an employee to attain the goal of that particular activity, it will lead to the allocation of less time than preferred to another activity and impede the attainment of any other goals. Consequently, these failures of goal attainment will decrease employee well-being. Thus:

Hypothesis 2a: There is an inverted U-shaped curvilinear relationship between self-discrepant time allocation for work activities that require more self-regulatory resources and employee well-being.
Hypothesis 2b: There is an inverted U-shaped curvilinear relationship between self-discrepant time allocation for work activities that require less self-regulatory resources and employee well-being.

#### Psychological Detachment, Self-Discrepant Time Allocation at Work, and Employee Well-Being

Previous studies have reported a positive relationship between psychological detachment and well-being indicators (Sonnentag and Fritz, 2015; Sonnentag et al., 2017). Theoretically, psychological detachment from work during non-work time helps employees to restore personal resources that were depleted in the preceding work period, which in turn promotes well-being (Fritz et al., 2010a). Prior empirical research has shown that psychological detachment reduces emotional exhaustion and negative affect (Hahn et al., 2011), and increases life satisfaction (Sonnentag and Fritz, 2007) and positive affect (Davidson et al., 2010). However, while such studies have demonstrated that psychological detachment is positively associated with well-being outcomes, the underlying mechanism of this relationship has largely been neglected in the research. Consequently, Sonnentag et al. (2017) suggested that future studies should explicitly address the mechanisms underlying the effects of recovery experiences on well-being. Given our postulations that psychological detachment relates to self-discrepant time allocation (Hypotheses 1a and 1b) and self-discrepant time allocation relates to employee well-being (Hypotheses 2a and 2b), we further posit that self-discrepant time allocation at work will mediate the positive relationship between detachment and employee well-being. More specifically, when psychological detachment is poor and self-regulatory resources are insufficient, employees will find it more difficult to allocate time in accordance with their preferences at work. This discrepancy will further decrease employee well-being. Thus:

Hypothesis 3: Psychological detachment is positively related to employee well-being.
Hypothesis 4a: Self-discrepant time allocation in respect to work activities that require more self-regulatory resources will mediate the effect of psychological detachment on employee well-being.
Hypothesis 4b: Self-discrepant time allocation in respect to work activities that require less self-regulatory resources will mediate the effect of psychological detachment on employee well-being.

## Materials and Methods

### Participants and Procedures

We collected data from faculty members working in 19 different universities in Guangdong province in China. A faculty sample is ideal to use to verify our hypotheses, for two reasons. First, previous studies have established that faculty work can be categorized into research, teaching, and service activities (Dahm et al., 2015). Second, faculty members have a high degree of job control; specifically, they have the discretion to allocate time to different work activities.

This study is part of a larger research project. With the help of Guangdong Educational, Scientific, Culture, and Health Industry Worker's Trade Union, 989 faculty members were recruited. All surveys were completed via wjx.com, a professional questionnaire survey site in China. According to our research framework, participants responded to two surveys at two different points within 1 week in October 2017. The first survey was administered on a Monday (at 9 a.m. GMT + 8) and assessed psychological detachment from the preceding weekend as well as demographic characteristics (e.g., gender, academic rank, administrative duties, marital status, spouse employment). The second survey was administered on a Friday (at 6 p.m. GMT + 8), and measured time allocations at work (e.g., actual and preferred time allocations for research, teaching, service, and administration activities), workplace well-being, and hours worked during the past week's workdays. Participants were allowed to submit their data only if they reported their cell phone number at the start of each survey. This procedure allowed us to match the surveys. We received 584 completed surveys, for a response rate of 59%. This response rate is favorable for this particular employee group.

We filtered the sample according to three criteria. First, responses to bogus items embedded within the surveys (e.g., “I want you to select ‘Disagree”) had to be correct. Second, responders had to be tenured or tenure-track faculty, since their work usually includes teaching, research, and service activities. Third, responders' actual and preferred time allocations need to have totaled 100%. Our final sample of 390 participants included 132 men (33.8%) and 258 women (66.2%); 78 participants were single (20%), 312 participants were married (80%). The distribution of academic rank was 139 full professors (35.6%), 140 associate professors (35.9%), 80 assistant professors (20.5%), and 31 lecturers (7.9%); 121 (31%) participants had administrative appointment at that time. The average working time in that week was 47.48 h (*SD* = 11.54).

### Measures

#### Psychological Detachment

Psychological detachment during the weekend was measured with four items from the Recovery Experience Questionnaire (Sonnentag and Fritz, 2007). Each item was answered on a 6-point Likert scale ranging from 1 (*I do not agree at all*) to 6 (*I fully agree*). Items included “During the weekend, I forgot about work” and “During the weekend, I didn't think about work at all.” Cronbach's alpha was 0.92.

#### Self-Discrepancy time Allocations at Work

Based on the faculty context, we conceptualized that work activities would be divided into four categories: research, teaching, service, and administrative duties (Dahm et al., 2015). We assessed self-discrepancy time allocations at work in two steps. First, we measured time allocations using items from Dahm et al. (2015). More precisely, to measure actual time allocation, participants were asked: “Please indicate what percentage of your work time you spend on teaching, research, service, and administrative duties during this week.^2^ Please ensure that your indicated percentages total 100%.” To measure preferred time allocation, participants were asked a parallel item: “Please indicate what percentage of your work time you would prefer to spend on teaching, research, service, and administrative duties during this week. Please ensure that your indicated percentages total 100%.” Descriptions of each category were provided.

Second, we operationalized self-discrepant time allocation in two ways to test our hypotheses. Firstly, activity-specific (i.e., teaching, research, and service) self-discrepant time allocations were operationalized as the difference between the preferred and actual percentage time allocations in each activity (i.e., preferred–actual) (Winslow, 2010; Liss et al., 2013; Dahm et al., 2015). Positive values suggested that actual time was less than preferred, while negative values suggested actual time was greater than preferred. Secondly, total self-discrepant time allocation was calculated as the sum of the absolute value of the discrepancies in each activity. Activity-specific and total self-discrepant time allocation scores were used to test Hypothesis 1a/1b, Hypothesis 3 and Hypothesis 4a/4b. In addition, we used quadratic models in actual and preferred time allocations, plus the interaction between actual and preferred time allocations, for the test of Hypothesis 2a/2b through polynomial regressions and response surface methodology (Edwards and Parry, 1993).

### Employee Well-Being

Employee well-being at work was assessed with the six-item workplace well-being subscale of the Employee well-being Scale, since it illustrates individuals' perceptions of their well-being in the workplace (Zheng et al., 2015). Items were scored on a 6-point Likert scale (1 = *strongly disagree*, 6 = *strongly agree*). Items included “This week, work is a meaningful experience for me” and “This week, I am satisfied with my work responsibilities.” Cronbach's alpha was 0.93.

### Controls

We controlled for an array of personal and work-related variables that have been proven to influence our predictor and outcome variables. Work-related factors included academic ranks (1 = *full professor*, 2 = *associate professor*, 3 = *assistant professor*, 4 = *lecturer*), administrative duties, and hours worked. Administrative data were assessed with the question: “At this stage, do you have an administrative appointment?” (1 = *yes*, 2 = *no*). Hours worked were ascertained by asking, “How many hours did you work this week?” Personal factors included gender (1 = *male*, 2 = *female*), marital status (1 = *single*, 2 = *married*, 3 = *other*), and spouse employment status (1 = *employed*, 2 = *not employed*).

Since all participants of our study were Chinese, we followed a double-blind back-translation process to translate the questionnaires cited above into Chinese. Each item was translated by professional translators to obviate translation ambiguity.

### Data Analysis

It was possible that the data used in the current study was nested, as we collected it from 390 participants belonging to 19 universities. However, we expected that it would be individual differences, rather than university differences, that would account for variance among the variables. Furthermore, in accordance with a recommendation given in LeBreton and Senter (2008), we divided the total variance into within-group and between-group variance to examine whether our variables differed substantially between groups (universities). As shown in Table 1 (below), for workplace well-being (1.82%, *p* > 0.05), self-discrepant total time (0.87%, *p* > 0.05), self-discrepant research time (1.95%, *p* > 0.05), self-discrepant teaching time (1.67%, *p* > 0.05), self-discrepant service time (0.62%, *p* > 0.05), and psychological detachment (0.01%, *p* > 0.05) between-groups variance was not significant and only accounted for a small part of total variance. The group effect was not an important factor in our study, which suggests that ordinary least squares regression modeling was appropriate for data analysis.

**Table 1:** Variance components of all variables.

	**Detachment**	**Self-discrepant total time**	**Self-discrepant research time**	**Self-discrepant teaching time**	**Self-discrepant service time**	**Workplace well-being**
Between-groups variance	0.00017	0.00063	0.00027	0.00039	0.00027	0.01325
Total variance	1.67301	0.07213	0.01379	0.02325	0.04385	0.7265
Between-groups/total (%)	0.01	0.87	1.95	1.67	0.62	1.82
*p*	0.801	0.158	0.174	0.325	0.158	0.31

*N = 390. p-value represents the significant of chi-square test for between-group variance*.

We tested our hypotheses following several steps. First, we used linear regression to test Hypothesis 3. Second, we followed the procedures recommended in Edwards (1995) to test Hypothesis 1a/1b. We used the difference scores as dependent variables, and also tested the relationships between detachment and actual time allocation and preferred time allocation separately. This addressed concerns that computing a composite difference score from two variables would lose information (Edwards and Parry, 1993; Bono and Judge, 2003). Third, we examined Hypotheses 2a/2b by the means of polynomial regression and response surface methodology, to gain insight into these relationships (Edwards and Parry, 1993). Polynomial regression can generate three-dimensional response surfaces, examining the congruence/incongruence effects (e.g., fit, match, similarity, agreement) on outcomes.

Specifically, workplace well-being (*Z*) was regressed on control variables as well as five polynomial terms; that is, actual time allocation (*A*), preferred time allocation (*P*), actual time allocation squared (*A*^2^), the interaction between actual and preferred time allocations (*A* × *P*), and preferred time allocation squared (*P*^2^).^3^ We centered variables to reduce collinearity in the higher-order variables. Thus:

$$\begin{array}{l} \text{Z}\;=\;{\text{b}}_{0}+{\text{b}}_{1}\text{A}+{\text{b}}_{2}\text{P}+{\text{b}}_{3}{\text{A}}^{2}+{\text{b}}_{4}\left(\text{A}\times \text{P}\right)+{\text{b}}_{5}{\text{P}}^{2}+\text{e} \end{array}$$

First, the significance of the second-order terms (*A*^2^, *A* × *P, P*^2^) was jointly tested, which is a prerequisite for three-dimensional response surface analysis. Second, a congruence effect was examined, based on the significance of the curvature along the incongruence line (*P* = –*A*). A significantly negative value of the curvature along the incongruence line means that the surface along the incongruence is inverted U-shaped, such that workplace well-being (dependent variable, perpendicular horizontal axes) decreases when preferred and actual allocated time (independent variables, vertical axis) differ from each other in either direction. In addition, the congruence line (*P* = *A*) can be used to compare the effect of two combined predictors on the outcome variable when they are aligned at a higher level vs. at a lower level.

Third, the slope and intercept of the first principal axis were examined. For concave surfaces, the ridge that describes the peak of the surface should run along the congruence line (*A* = *P*), then the first principal axis of the surface should have a slope of 1 and an intercept of 0.95% confidence intervals (CIs) for the slope/intercept of first principal axis, with 10,000 bootstrapped samples used to assess the significance. If the CI for the intercept included a 0 and the CI for the slope included a 1, we could conclude that the shape was symmetrical, which means that the congruence point (the point across the congruence line and incongruence line) is the highest/lowest point of the outcome. On the contrary, if the CI for the intercept did not involve a 0 and the CI for the slope did not include a 1, the shape would be asymmetrical, which means that the highest/lowest point was not the congruence point. Additionally, the slope along the congruence line could also be tested. This value would determine whether the outcome would be different when two combined predictors were aligned at a higher level vs. at a lower level. However, given that this study's focus was mainly on the effects of disunity between actual time allocation and preferred time allocation, the significance of slope along the congruence line was not tested. The results are available upon request from the first author.

Finally, as it is unconventional to test for mediation using polynomial variables, we used difference scores of self-discrepancies to test our mediation hypotheses (Edwards and Lambert, 2007; Colbert et al., 2008; Dahm et al., 2015).

## Results

### Description

Table 2 shows the means, standard deviations, and correlations of the variables. Detachment is shown to be significantly correlated with self-discrepant time allocation for total (*r* = −0.15, *p* < 0.01), research (*r* = −0.21, *p* < 0.01), teaching (*r* = 0.20, *p* < 0.01), and workplace well-being (*r* = 0.16, *p* < 0.01) results, but not for service (*r* = −0.08, *p* > 0.05).

**Table 2:** Descriptive statistics and correlations among all variables.

	***M***	***SD***	**1**	**2**	**3**	**4**	**5**	**6**	**7**	**8**	**9**	**10**	**11**	**12**	**13**	**14**	**15**	**16**	**17**
1. Gender	1.66	0.47	—															
2. Academic rank	2.01	0.94	0.01	—														
3. Administrative duties	1.68	0.47	0.01	−0.09	—													
4. Marital status	1.80	0.40	0.09	0.11^*^	−0.04	—												
5. Spouse employment	1.13	0.34	−0.04	−0.05	0.07	−0.18^**^	—											
6. Hours worked	47.48	11.54	−0.17^**^	−0.02	0.01	−0.03	0.07	—										
7. Detachment	3.72	1.29	−0.03	−0.03	−0.02	0.01	−0.05	−0.05	—									
8. Total time (self-discrepant)	0.42	0.27	−0.02	−0.10^*^	0.33^**^	0.04	−0.03	−0.02	−0.15^**^	—								
9. Research time (self-discrepant)	0.05	0.12	0.11^*^	−0.03	0.05	0.09	−0.09	−0.03	−0.21^**^	0.51^**^	—							
10. Actual research time	0.22	0.11	−0.10^*^	0.12^*^	−0.02	−0.11^*^	−0.02	−0.10^*^	0.12^*^	−0.39^**^	−0.57^**^	—						
11. Preferred research time	0.27	0.11	0.02	0.10	0.03	−0.02	−0.10	−0.14^**^	−0.11^*^	0.15^**^	0.51^**^	0.42^**^	—					
12. Teaching time (self-discrepant)	0.01	0.15	−0.09	−0.04	−0.01	−0.01	0.10	0.01	0.20^**^	0.24^**^	−0.27^**^	0.11^*^	−0.18^**^	—				
13. Actual teaching time	0.31	0.16	0.11^*^	0.03	−0.04	0.01	−0.12^*^	−0.07	−0.11^*^	−0.26^**^	0.15^**^	−0.16^**^	−0.04	−0.72^**^	—			
14. Preferred teaching time	0.32	0.12	0.03	−0.01	−0.07	0.01	−0.04	−0.08	0.11^*^	−0.06	−0.14^**^	−0.08	−0.24^**^	0.27^**^	0.46^**^	—		
15. Service time (self-discrepant)	−0.18	0.21	0.05	0.12^*^	−0.50^**^	−0.04	−0.07	−0.05	−0.08	−0.67^**^	−0.26^**^	0.30^**^	0.03	−0.57^**^	0.52^**^	−0.01	—	
16. Actual service time	0.40	0.16	−0.05	−0.14^**^	0.53^**^	0.05	0.12^*^	0.09	0.04	0.60^**^	0.18^**^	−0.37^**^	−0.19^**^	0.47^**^	−0.66^**^	−0.32^**^	−0.85^**^	—
17. Preferred service time	0.22	0.11	0.01	−0.03	0.04	0.02	0.08	0.07	−0.08	−0.14^**^	−0.14^**^	−0.12^*^	−0.27^**^	−0.20^**^	−0.24^**^	−0.59^**^	0.29^**^	0.26^**^	—
18. Workplace well-being	4.38	0.85	−0.11^*^	−0.01	−0.10	0.02	−0.06	−0.05	0.16^**^	−0.32^**^	−0.13^**^	0.07	−0.07	−0.08	0.12^*^	0.07	0.14^**^	−0.17^**^	−0.06

*N = 390*.

* *p < 0.05*.

** *p < 0.01*.

### Confirmatory Factor Analyses and Discriminant Validity

We performed confirmatory factor analyses to examine the discriminant validity of psychological detachment and workplace well-being. The results showed a better fit for the two-factor model, χ^2^ = 70.22, *df* = 34, *p* < 0.001, RMSEA = 0.05, CFI = 0.98, GFI = 0.97, NFI = 0.97, SRMR = 0.04 (Hu and Bentler, 1999), with all items loading on their corresponding factors, than for a one-factor model, χ^2^ = 1055.37, *df* = 35, *p* < 0.001, RMSEA = 0.27, CFI = 0.55, GFI = 0.53, NFI = 0.54, SRMR = 0.32. The chi-square difference also reached a significant level, Δχ^2^(1) = 28.09, *p* < 0.001. Thus, psychological detachment and workplace well-being could be distinguished at the construct level. We didn't include time allocation variables in the confirmatory factor analyses as these variables were measured as indices of time spent on work activities and could no longer be considered as a latent variable (construct), but rather as an observed variable. This approach is often used to conduct confirmatory factor analyses in recovery studies (in which recovery activities are measured as time spent on the activity) (Sonnentag and Natter, 2004; Mojza et al., 2011).

### Common Method Bias

Since the data of the current study were gathered through self-report measures, common method bias could inflate the perceived relationships (Podsakoff et al., 2003). We conducted two tests to examine whether common method bias was a problem in respect to the present study. First, we used Harman's single-factor test to assess the common method bias. The result of the unrotated factor solution showed that the “general” single factor only explains 26.17% of the variance in the variables. Second, we performed the common latent factor approach to further examine the common method bias. These results showed that adding a common method factor did not increase the mode fit significantly, Δχ^2^(16) = 0.15, *p* > 0.05. Thus, common method bias was not found to be a serious problem in the present study.

### Hypothesis Testing

Hypotheses 1a and 1b predicted that psychological detachment would be negatively related to self-discrepant time allocation. That is, time allocation would become less discrepant when psychological detachment increased. Results from our study's regression analyses show that psychological detachment negatively relates to total self-discrepant time allocation, *b* = −0.03, *p* < 0.001 (see Table 3).

**Table 3:** Regressions of detachment on total self-discrepant time and self-discrepant, actual, and preferred research, teaching time, and workplace well-being.

**Variables**	**Self-discrepant total time**		**Self-discrepant research time**		**Actual research time**		**Preferred research time**		**Self-discrepant teaching time**		**Actual teaching time**		**Preferred teaching time**		**Workplace Well-being**	
	**Step 1**	**Step 2**	**Step 1**	**Step 2**	**Step 1**	**Step 2**	**Step 1**	**Step 2**	**Step 1**	**Step 2**	**Step 1**	**Step 2**	**Step 1**	**Step 2**	**Step 1**	**Step 2**
Constant	0.19	0.34^**^	0.01	0.08	0.35^***^	0.31^***^	0.34^***^	0.39^***^	0.02	−0.09	0.38^***^	0.44^***^	0.40^***^	0.35^***^	5.40^***^	4.93^***^
Gender	−0.02	−0.03	0.03^†^	0.02^†^	−0.03^*^	−0.02^*^	−0.01	−0.01	−0.03^†^	−0.03	0.03^†^	0.03^†^	0.01	−0.01	−0.23^*^	−0.22^*^
Academic ranks	−0.02^†^	−0.02^†^	−0.01	−0.01	0.02^**^	0.02^**^	0.01^†^	0.01^†^	−0.01	−0.01	0.01	0.01	−0.01	−0.01	−0.02	−0.02
Administrative duties	0.19^***^	0.19^***^	0.01	0.01	−0.01	−0.01	0.01	0.01	−0.01	−0.01	−0.01	−0.01	−0.02	−0.02	−0.18^†^	−0.18^†^
Marital status	0.04	0.04	0.02	0.02	−0.03^*^	−0.03^*^	−0.01	−0.01	0.01	0.01	−0.01	−0.01	−0.01	−0.01	0.05	0.05
Spouse employment	−0.03	−0.04	−0.03	−0.03	−0.01	−0.01	−0.03^†^	−0.03^†^	0.04^†^	0.05*	−0.06^*^	−0.06^*^	−0.01	−0.01	−0.14	−0.12
Hours worked	−0.01	−0.01	−0.01	0.01	−0.01^*^	−0.01^*^	−0.01^*^	−0.01^**^	0.01	0.01	−0.01	−0.01	−0.01	−0.01	−0.01	−0.01
Detachment		−0.03^**^		−0.02^***^		0.01*		0		0.02^***^		−0.01^*^		0.01^†^		0.11**
*R*^2^	0.12^***^	0.15^***^	0.03	0.07^***^	0.05^**^	0.06^**^	0.04^*^	0.04^*^	0.02	0.06^**^	0.03^†^	0.04^*^	0.01	0.02	0.18^†^	0.24^**^
Δ*R*^2^		0.03^**^		0.04^***^		0.01^*^		0		0.04^***^		0.01^*^		0.01^†^		0.06^**^

N = 390. Coefficients are unstandardized.

† p < 0.10.

* p < 0.05.

** p < 0.01.

*** *p < 0.001*.

Furthermore, Hypothesis 1a/1b assumed that actual time allocated to work activities requiring high levels of self-regulatory resources (i.e., research activities) would be more than preferred time as psychological detachment increased, and that actual time allocated to work activities requiring low levels of self-regulatory resources (i.e., teaching and service activities) would be less than preferred time. Our results indicate that psychological detachment negatively relates to self-discrepant research time allocation, *b* = −0.02, *p* < 0.001 (see Table 3), and positively relates to self-discrepant teaching time allocation, *b* = 0.02, *p* < 0.001 (see Table 3). However, psychological detachment does not relate to self-discrepant service time, *b* = −0.004, *p* > 0.05. Therefore, Hypotheses 1a and 1b were only partially supported.

In addition, we regressed actual time allocations on psychological detachment after controlling for preferred time allocations to make our interferences more robust (Scott and Barnes, 2011). The results show that psychological detachment positively relates to actual research time allocation, *b* = 0.01, *p* < 0.05 (see Table 3), and negatively relates to actual teaching time allocation, *b* = −0.01, *p* < 0.05 (see Table 3), but not actual service time allocation, *b* = 0.008, *p* > 0.05 (see Table 3). These results provide further evidence that psychological detachment relates to self-discrepant time allocation in research and teaching domains.

**Figure 1:**
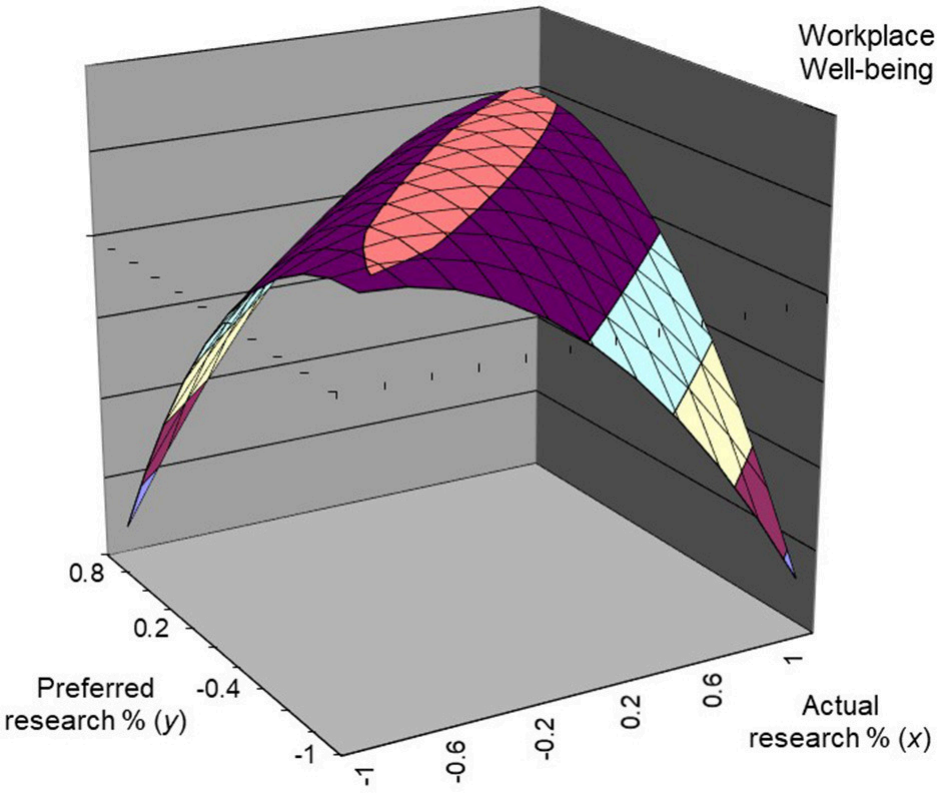
Effects of actual and preferred research time allocation on workplace well-being.

Hypotheses 2a and 2b proposed that employee well-being would be higher when actual time allocation was congruent with preferred time allocation than when they were incongruent. Through our analysis, we found that total self-discrepancy negatively relates to workplace well-being, *b* = −1.08, *p* < 0.001. Tables 4–6 show the results of the polynomial regressions of the relationships between research, teaching, and service time allocations and workplace well-being. For research time allocation, the addition of the three second-order polynomial terms jointly changes the *R*-squared value in the regression equations for workplace well-being, *R*^2^ = 0.02, *p* < 0.05, and the curvature along the incongruence line was negative and significant (curvature = −22.84, *p* < 0.01; see Table 4). Figure 1 illustrates the response surface based on regression coefficients in Table 4. The incongruence line was from the left corner to the right corner. The negative curvature along the incongruence indicated that the surface progressed downward. That is, it was an inverted U-shaped surface along the incongruence line. As the curvature was negative, we calculated the first principal axes, and found that the value of the intercept did not differ significantly from 0, 95% CI [−0.23, 0.01], and that of the slope did not differ significantly from 1, 95% CI [0.42, 1.52]. These results indicate that well-being had the greatest value at the congruence point, supporting the supposition that, when actual research time allocation and preferred research time allocation are not aligned, employees' well-being decreases.

**Table 4:** Polynomial regressions of workplace well-being on research time allocation.

**Variables**	Workplace well-being		
	**Step 1**	**Step 2**	**Step 3**
Constant	5.39^***^	5.36^***^	5.46^***^
Gender	−0.24^*^	−0.22^*^	−0.22^*^
Academic ranks	−0.02	−0.02	−0.02
Administrative duties	−0.17^†^	−0.16^†^	−0.16^†^
Marital status	0.05	0.07	0.05
Spouse employment	−0.14	−0.17	−0.17
Hours worked	−0.01	−0.01	−0.01
Actual research time (*A*)		0.83^†^	0.57
Preferred research time (*P*)		−1.01^*^	−0.46
*A*^2^			−5.49^*^
*A* × *P*			11.19^**^
*P*^2^			−6.16^*^
*R*^2^	0.03^†^	0.05^*^	0.07^**^
Δ*R*^2^		0.02^*^	0.02^*^
Congruence line (*P* = *A*)
Slope	−0.46
Curvature	−0.94
Incongruence line (*P* = –*A*)
Slope	1.03
Curvature	−22.84^**^

N = 390. Coefficients are unstandardized.

† p < 0.10.

* p < 0.05.

** p < 0.01.

*** *p < 0.001*.

**Table 5:** Polynomial regressions of workplace well-being on teaching time allocation.

**Variables**	Workplace well-being		
	**Step 1**	**Step 2**	**Step 3**
Constant	5.40^***^	5.35^***^	5.58^***^
Gender	−0.23^*^	−0.26^**^	−0.27^**^
Academic ranks	−0.02	−0.02	−0.04
Administrative duties	−0.18	−0.17	−0.12
Marital status	0.05	0.06	0.04
Spouse employment	−0.14	−0.11	−0.15
Hours worked	−0.01	−0.01	−0.01
Actual teaching time (*A*)		0.62^*^	0.80^*^
Preferred teaching time (*P*)		−0.06	−0.22
*A*^2^			−4.40^***^
*A* × *P*			12.552^***^
*P*^2^			−9.19^***^
*R*^2^	0.03^†^	0.05^†^	0.11^**^
Δ*R*^2^		0.02^†^	0.06^***^
Congruence line (*P* = *A*)
Slope	0.58
Curvature	−1.04
Incongruence line (*P* = –*A*)
Slope	1.02
Curvature	−26.14^***^

*N = 390. Coefficients are unstandardized*.

† p < 0.10.

* p < 0.05.

** p < 0.01.

*** *p < 0.001*.

**Table 6:** Polynomial regressions of workplace well-being on service time allocation.

**Variables**	Workplace well-being		
	**Step 1**	**Step 2**	**Step 3**
Constant	5.39^***^	5.02^***^	5.38^***^
Gender	−0.24^*^	−0.25^**^	−0.26^**^
Academic ranks	−0.02	−0.04	−0.04
Administrative duties	−0.17^†^	−0.01	−0.10
Marital status	0.05	0.09	0.11
Spouse employment	−0.14	−0.10	−0.12
Hours worked	−0.01	−0.01	−0.01
Actual service time (*A*)		−0.11	−0.37
Preferred service time (*P*)		−0.06	−0.15
*A*^2^			−2.45^**^
*A × P*			0.68
*P*^2^			−1.45
*R*^2^	0.03^†^	0.05^**^	0.07^**^
Δ*R*^2^		0.02^*^	0.02^*^
Congruence line (*P* = *A*)
Slope	−0.52
Curvature	−4.58
Incongruence line (*P* = –*A*)
Slope	−0.22
Curvature	−4.58

N = 390. Coefficients are unstandardized.

† p < 0.10.

* p < 0.05.

** p < 0.01.

*** *p < 0.001*.

**Figure 2:**
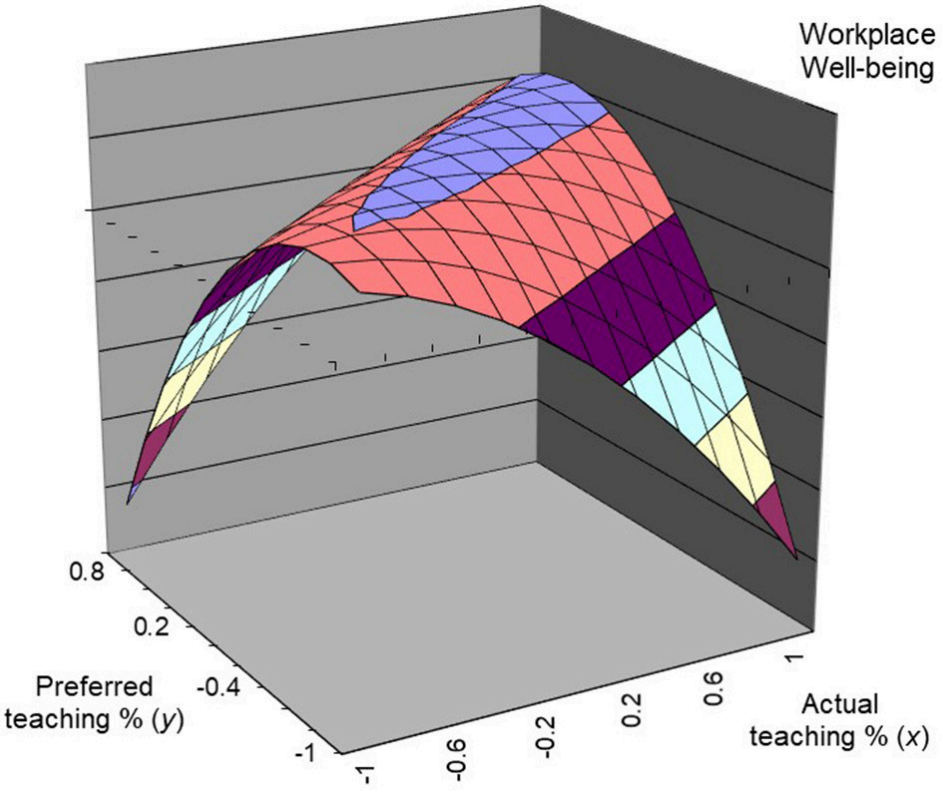
Effects of actual and preferred teaching time allocation on workplace well-being.

Meanwhile, for teaching time allocation, the addition of the three higher-order variables significantly changed the *R-squared* value in the regression equation for workplace well-being, *R*^2^ = 0.06, *p* < 0.001, and the curvature along the line of incongruence was negative and significant (curvature = −26.14, *p* < 0.001; see Table 5). Figure 2 illustrates the response surface. The incongruence line and congruence line proved to be the same as research time allocation, and an inverted U-shaped surface along the incongruence line was also found. The value of the intercept did not differ significantly from 0, 95% CI [−0.08, 0.01], and slope value was not significantly different from 1, 95% CI [0.46, 1.02], indicating that well-being had the greatest value at the congruence point, supporting the postulation that, when actual teaching time allocation and preferred teaching time allocation were not aligned, employees' well-being decreased.

For service time allocation, the addition of the three higher-order variables significantly changed the *R-squared* value in the regression equation for workplace well-being, *R*^2^ = 0.02, *p* < 0.05, but the curvature along the incongruence line was not significant (curvature = −4.58, *p* > 0.05; see Table 6). Therefore, the congruence effect was found to exist for research and teaching time allocations, but not for service time allocation. In sum, the congruence effect exists for research and teaching time allocations, but not for service time allocation. Hypothesis 2a was fully supported and Hypothesis 2b was partially supported.

Hypothesis 3 predicted that psychological detachment would be positively related to employee well-being. Through our analysis, we found that psychological detachment positively relates to workplace well-being, b = .11, *p* < 0.01. Hypothesis 3 was fully supported.

Hypotheses 4a and 4b predicted that self-discrepancy would mediate the relationship between detachment and workplace well-being. We examined the mediation effect by using path analysis (Preacher and Hayes, 2008; Dahm et al., 2015), which provided the direct, indirect, and total effects of psychological detachment on workplace well-being, as well as the significance of the indirect effects, by using 10,000 bootstrap samples to construct 95% CIs and testing the significance of the indirect effects (Edwards and Lambert, 2007). As shown in Table 7, the indirect effect of psychological detachment, via total time allocation, is significant for workplace well-being [0.031; 95% CI (0.0111, 0.0527)]. In the research domain, the indirect effect of psychological detachment through self-discrepant research time allocation is significant for workplace well-being [0.014; 95% CI (0.0005, 0.0310)]. In the teaching domain, the indirect effect of psychological detachment through self-discrepant teaching time allocation is also significant for workplace well-being [−0.016; 95% CI (−0.0359, −0.0012)]. However, self-discrepant service time does not mediate the effects of psychological detachment on workplace well-being [−0.008; 95% CI (−0.0225, 0.0010)].

**Table 7:** Path analysis: indirect and total effects of psychological detachment via total, research, teaching, and service time allocations on workplace well-being.

**Paths**	**P_MX_**	**P_YM_**	**Direct effects (P_YX_)**	**Indirect effects (P_YM_ × P_MX_)**	**Total effects [P_YX_ + (P_YM_ × P_MX_)]**
Detachment → Total → Workplace well-being	−0.031^**^	−0.971^***^	0.074^*^	0.031^*^	0.105^**^
			95% CI (0.0304, 0.0067)	95% CI (0.0111, 0.0527)	
Detachment → Research → Workplace well-being	−0.019^***^	−0.758^*^	0.091^**^	0.014^*^	0.105^**^
			95% CI (0.0081, 0.0237)	95% CI (0.0005, 0.0310)	
Detachment → Teaching → Workplace well-being	0.024^***^	−0.645^*^	0.121^***^	-0.016^*^	0.105^**^
			95% CI (0.0004, 0.0538)	95% CI (−0.0359, −0.0012)	
Detachment → Service → Workplace well-being	−0.014^†^	0.623^**^	0.113^***^	−0.009	0.104^**^
			95% CI (0.0008, 0.0471)	95% CI (-0.0225, 0.0010)	

N = 390. Coefficients are unstandardized.

† *p < 0.10*.

* p < 0.05.

** p < 0.01.

*** *p < 0.001*.

## Discussion

Increasing numbers of employees are continually thinking about or dealing with job tasks during off-job time in order to achieve better performance and career success. However, it remains unclear how this approach affects the behavior and feelings of these employees in the subsequent work period. Specifically, it is far from fully understood how psychological detachment during leisure time affects work behavior and well-being in subsequent work periods. Based on self-regulation theory, we hypothesized and found that, when psychological detachment during a weekend is low and self-regulatory resources are insufficient, employees will allocate less time than preferred to work activities that require more self-regulatory resources (i.e., researching activities) during the following work period. Instead, these employees tend to allocate more time to activities that require less resources (i.e., teaching activities). Such self-discrepancy in time allocation was, in turn, identified as affecting employee well-being. Further, these self-discrepant time allocations were found to mediate the relationship between psychological detachment and employee well-being.

### Theoretical implications

The present study extends existing research in the detachment literature in a number of important ways. First, by suggesting that psychological detachment predicts time allocation behavior at work, we extend knowledge regarding the influence of psychological detachment on working behavior. Prior research has mainly focused on the effects of psychological detachment on general well-being indicators and strain reactions (Sonnentag and Fritz, 2015). For example, studies have shown that psychological detachment is negatively related to emotional exhaustion, negative affect, and psychological strain and is positively related to life satisfaction, sleep quality, and work engagement (Moreno-Jiménez et al., 2009; Hahn et al., 2011). Although employees and organizations may be more concerned about changes in work behavior caused by different levels of psychological detachment, only a few studies have investigated these linkages (Binnewies et al., 2010; Fritz et al., 2010a). Sonnentag (2018) suggested that more research is needed that addresses the question of how psychological detachment relates to various dimensions of work behavior. Focusing on time, which is a critical element of work, our study investigated how psychological detachment affects time spent on different work activities. Our findings demonstrate that disconnecting from work-related issues helps employees to replenish self-regulatory resources and consequently to apply them to complex and long-term tasks (e.g., research activities), rather than tasks that offer immediate rewards and are easier to complete (e.g., teaching activities). These results are the first to suggest that psychological detachment may change how employees apportion their time among different work activities. In this way, our study fills the gaps in comprehension of the relationships between psychological detachment and work behavior.

Second, we consider the mechanism underlying the impact of detachment on well-being by verifying the mediating effect of self-discrepant time allocation at work. Previous studies have mainly concentrated on the direct impact of psychological detachment on general well-being indicators. The mechanism behind this association, though, has been largely neglected. While it makes sense to explore the direct effect first and to address the underlying mechanism later, as stressed by Sonnentag et al. (2017), “Future studies should explicitly address the mechanisms underlying the effects of recovery experiences on well-being” (p. 373). We respond to this call by demonstrating a work-related behavioral mediating mechanism. Specifically, when employees experience lower detachment during a weekend and self-regulatory resources are low, they will allocate less time than preferred to complex and long-term goal activities (e.g., research activities) and allocate more time than preferred to easier and short-term goal activities (e.g., teaching activities). This discrepancy, in turn, will decrease employees' workplace well-being. For the first half of these paths, the notion that psychological detachment may influence time allocation at work is unique and provides evidence for the replenishment effect of psychological detachment on self-regulatory resources. Previous studies have theoretically inferred that psychological detachment facilitates restoration in self-regulatory resources because it prevents a further loss of depleted self-regulatory resources and provides an opportunity to gain a positive mood, as described by the conservation of resources theory (Sonnentag and Fritz, 2015). In recent years, a few studies have provided indirect empirical evidence for this inference (Rivkin et al., 2015; Germeys and De Gieter, 2018). For example, Germeys and De Gieter (2018) found that psychological detachment is directly negatively related to ego depletion experienced at home. Since ego depletion can be seen as a proxy indicator that reflects a lack of regulatory resources, this result provides indirect evidence for the replenishment effect of psychological detachment on self-regulatory resources. Similarly, self-discrepant time allocation at work can also be seen as a proxy indicator of self-regulatory resources, as, to a certain extent, self-regulatory resources affect whether an individual can allocate time according to their preferences, goals, and wishes (Dahm et al., 2015). Thus, our results also verify the replenishment effect of psychological detachment on self-regulatory resources. For the second half of these paths, although the use of self-discrepant time allocation at work as an explanation mechanism is entirely new in the psychological detachment literature, prior research has indicated that there are inverted U-shaped curve relationships concerning the discrepancies between actual and preferred time allocations and work activities and well-being indicators. For example, studies have shown job stress and work-family conflict are higher for employees experiencing a conflict between actual and preferred work hours, regardless of whether actual work hours are less or more than preferred (Sturman and Walsh, 2014); moreover, work satisfaction and physical well-being have been found to be lower for employees with misaligned time allocation, no matter whether the differences are positive or negative (Dahm et al., 2015).

In addition, we provide further evidence for the effect of detachment on employee well-being. Most research offers indirect evidence for the relationship between detachment and employee well-being by examining the influence of detachment on general well-being indicators (e.g., life satisfaction, positive and negative affects, psychological strain). Nevertheless, overall well-being cannot stand for well-being within the workplace since the workplace context differs greatly from that of general life situations. Some researchers have suggested that future studies should use context-specific measures to precisely capture employees' feelings and experiences at work (Zheng et al., 2015). We responded to this call by directly measuring employees' workplace well-being and examining the relationship between detachment and workplace well-being.

For activities requiring low self-regulatory resources, while service work activities were more discretionary than teaching work activities, our results show that psychological detachment did not significantly predict self-discrepant service time allocation. One potential reason for this finding is that service activities may demand more self-regulatory resources than teaching. The courses that a faculty member teaches are relatively fixed for years, and, thus, the faculty member is usually familiar with these courses. Conversely, service activities comprise a wide range of tasks (e.g., reviewing papers, organizing conferences, consulting for different organizations), and the scope and nature of service activities are relatively unpredictable and changeable. Accordingly, compared to teaching activities, with which faculty members are typically familiar, additional self-regulation resources are needed for service activities, since the complexity and ambiguity of these activities are higher. Thus, when a faculty member experiences low psychological detachment and depleted self-regulation resources, they may tend to allocate more time to activities requiring fewer self-regulatory resources, which, in our study, are teaching activities rather than service activities. For example, a depleted faculty member will likely be more willing to review the PowerPoint of a course that has been taught over several years, instead of reviewing a new manuscript sent by the editor of journal. Our results also show that self-discrepant service time allocation does not significantly predict workplace well-being. For some faculty members, the universities and departments for which they work do not regard service activities as the main reference points for evaluation and rewards. Some faculty members do not receive sufficient feedback from the workplace itself corresponding to the time and resources devoted to service activities. Accordingly, time allocation to service work is not related to workplace well-being.

### Practical Implications

When employees experience high psychological detachment and restore self-regulatory resources, they can deploy sufficient time to work activities that require more self-regulatory resources. This is beneficial for both employees and organizations. When the actual allocated time becomes consistent with their preferences regarding the same, an employee will perform better in goal attainment, and the “ideal self” of this employee at work, which reflects their goals, expectations, and wants, can thereby be better fulfilled. Consequently, employee well-being is improved. Since activities that demand greater self-regulatory resources are usually important and valuable, deploying plenty of time to these activities is particularly favorable (Dahm et al., 2015). Therefore, an organization and its employees should facilitate the latter's psychological detachment to ensure that they can allocate enough time to work activities, especially to activities that require additional self-regulatory resources. More precisely, an organization should cultivate its culture and develop norms to ensure that employees are not disturbed by work-related matters during leisure time. For example, supervisors should avoid sending e-mails and calling after work, as well as prevent the formation of expectations regarding availability and an always-on culture, to ensure that employees can disengage from work during off-job time (Derks et al., 2014, 2015). Equally, employees should engage in leisure activities that facilitate detachment during non-work time. These activities can include physical exercise, social activities with one's friend or partner, or developing a new hobby (Fritz et al., 2010b; Sonnentag, 2012). Also, employees, especially those who have difficulty with detaching from work during off-job time, can create a plan that focuses on specific, small goals for unfinished work, in order to enhance psychological detachment (Smit, 2016).

Although psychological detachment has been established as having various favorable effects, overworking nevertheless seems to be universal for today's employees. Previous studies have shown that low detachment decreases work engagement (Sonnentag and Fritz, 2015), yet our results show that low detachment does not necessarily weaken work engagement for all work activities. Employees tended to allocate more time than preferred in respect to activities that demanded high self-regulation (i.e., easy and short-term goal tasks) even when they had experienced low detachment. Accordingly, organizations should adjust job assignments in accordance with employees' preferences in order to make employees happier and more effective. That is, organizations should give employees who experience low detachment more opportunities to engage in low-demand activities. For example, organizations could reconfigure complex, long-term goals into tasks that are easier and more short-term, or, conversely, set the priority of low-demand tasks higher. Correspondingly, employees who experience low detachment could ensure that they finish certain tasks and feel fulfilled by autonomously adjusting their work process in terms of their resources and the demands of the work activities. For example, a depleted employee might prioritize simple and short-term tasks, or deploy more time to less complex and short-term tasks.

### Limitation and Future Research

Despite its theoretical and practical implications, the present study is subject to several limitations. One potential drawback lies with its design strategy. Although the two time points for assessment may lessen transient response biases, the cross-section design still limits inferences of causality. Future research should apply experience sampling methods or longitudinal designs in order to be able to draw more definitive conclusions about causality. In addition, our data were self-reported, which can cause self-report bias and ultimately contaminate a study's results (Podsakoff et al., 2012). Even though self-reporting is the only way to measure a person's psychological detachment and self-discrepant time allocations at work, it would be advantageous for future research to use some other measuring methods such as collecting objective data or coworker/supervisor assessment.

The current study was also limited by the composition of our sample, which consisted of faculty members at university. Typically, faculty members have a high degree of autonomy and can decide how to allocate their time among different work activities (Winslow, 2010). While the majority of occupations allow employees to control their time allocations to some degree, we recommend that future studies should examine the generalizability of our findings to employees in other occupations, especially employees who have less latitude regarding the regulation of time allocations according to their resource status, such as manufacturing factory workers and bank tellers.

Finally, as our research was focused on the mechanism underlying the effect of psychological detachment on employee well-being, we did not include moderating variables in this study. To obtain a more detailed picture of the relationship between psychological detachment and employee well-being, future research should consider moderators that might influence the association between detachment and time allocations at work or employee well-being. For example, individuals high in conscientiousness can be characterized as being hardworking, self-controlled, and persistent (Nandkeolyar et al., 2014); that is, highly conscientious employees are able to pursue long-term goals, overcome difficulties, and to persist in complex tasks even when the self-regulatory resources they held are less. Thus, conscientiousness may moderate the relationship between psychological detachment and self-discrepant time allocations at work.

## Ethics Statement

This study was carried out in accordance with the recommendations of ethics committee of Jinan University with written informed consent from all subjects. All subjects gave written informed consent in accordance with the Declaration of Helsinki. The protocol was approved by the ethics committee of Jinan University.

## Author Contributions

XW and AL designed the study. AL and PL collected the data. XW analyzed the results and wrote the manuscript. PL and MR revised the manuscript.

### Conflict of Interest Statement

The authors declare that the research was conducted in the absence of any commercial or financial relationships that could be construed as a potential conflict of interest.

## Footnotes

^1^“Preferred time” involves employee work goals and ideas, which are stable. Therefore, we suggest psychological detachment will affect actual time allocation, not preferred time allocation.

^2^Since not all faculty members had administrative responsibilities, but all had research, teaching, and service responsibilities, we did not include administration time in our analysis.

^3^For simplicity, we omitted all control variables, and both the actual and the preferred time allocation variables were scaled by 100 to decrease the range for the higher-order variables.
